# Association of the 2019 Canada’s food guide food choices assessment score with 10-year cardiovascular disease risk and heart age in Canadian adults

**DOI:** 10.1371/journal.pone.0331973

**Published:** 2025-10-22

**Authors:** Samer Hamamji, Daniel Zaltz, Mary R. L’Abbé

**Affiliations:** Department of Nutritional Sciences, Temerty Faculty of Medicine, University of Toronto, Toronto, Ontario, Canada; Makerere University College of Natural Sciences, UGANDA

## Abstract

Healthy diet plays an important role in the prevention of cardiovascular disease (CVD), which is the second leading cause of death in Canada. In 2019, Health Canada released an updated Canada’s Food Guide (CFG) which is accompanied with supportive evidence of Canada’s Dietary Guidelines (CDG) to reflect the latest evidence of the relationship between diet and prevention of chronic diseases including CVD. The Canadian Cardiovascular Society recommends the use of the Framingham risk score (FRS) to estimate the 10-year CVD risk and heart age in individuals aged 30 and older, aiding in CVD prevention interventions such as lifestyle modifications. However, the relationship between the intake of dietary choices aligned with 2019 CFG/CDG and CVD risk among Canadians was not studied. This study aims to examine the association between dietary choices assessed by a Food Choices Assessment Score (FCAS) according to 2019 CFG/CDG and 10-year CVD risk and heart age among Canadian adults. This cross-sectional study was conducted using the national Canadian Health Measures Survey (CHMS) (2016–2019) and included Canadian adults (≥ 30 years) without heart disease (n = 5,111). The 2019 CFG/CDG FCAS was calculated using the CHMS food frequency questionnaire. Canadians in the highest quintile (healthiest) of the FCAS had 55% lower odds (OR) of having high risk (≥ 20%) of estimated 10-year CVD risk (OR: 0.45; 95%CI: 0.23, 0.90) (*P*_trend_ = 0.011), and 47% lower odds of having unhealthy heart age difference (heart age > chronological age) (OR: 0.53; 95%CI: 0.30, 0.92) (*P*_trend_ = 0.02), compared to those in the first quintile (unhealthy) of the FCAS. This study indicates a strong inverse association of dietary choices as measured by the 2019 CFG/CDG FCAS with high 10-year CVD risk (≥ 20%) and unhealthy heart age (older than chronological age), estimated with the FRS.

## Introduction

Cardiovascular disease (CVD) is a significant public health concern in Canada, with approximately one in twelve adults diagnosed with the condition [1]. CVD is the second leading cause of the death, the leading cause of hospitalization, and contributes to a high economic burden [2–4]. Poor diet is a crucial risk factor that increases the burden of CVD-related deaths in Canada [5]. For example, in 2021, more than 32% of ischemic heart disease deaths were attributed to an unhealthy diet [6]. Evidence suggested that healthy dietary patterns, such as the Dietary Approaches to Stop Hypertension (DASH) and Mediterranean diets, play an important role in managing CVD risk factors and are linked with the reduction of CVD incidence and/or mortality [7,8]. The majority of CVD cases are preventable, and controlling risk factors such as poor diet can help reduce the growing burden of CVD on populations and health care systems [9–11]. Therefore, improving population’s diet is a key to the primary prevention of CVD. In 2019, Health Canada released an updated Canada’s Food Guide (CFG) as a part of the Healthy Eating Strategy that aims to improve population dietary intakes and prevent diet-related chronic diseases including CVD [12]. The 2019 CFG was accompanied by supportive evidence in Canada’s Dietary Guidelines (CDG) to reflect the latest evidence of the relationship between diet and prevention of chronic diseases [13,14].

The Framingham Risk Score (FRS) is a risk assessment tool developed based on the Framingham Heart Study which produced a revolution in understanding the prevention of CVD [15,16]. The FRS is a cluster of risk factors that was found to increase the risk of a CVD event, including age, sex, total cholesterol (TC), high density lipoprotein cholesterol (HDL-C), smoking habits, systolic blood pressure (SBP), and diabetes [15]. The FRS is used to estimate the 10-year CVD risk of individuals and determines their need for prevention strategies, such as lifestyle modifications and medical interventions [15]. To simplify communication of the CVD risk concept with individuals, the Framingham Heart Study introduced the estimation of heart age as a potential useful tool to motivate heart-healthy lifestyle changes [17]. It represents the age at which individual would have the same 10-year CVD risk of a healthy individual of the same sex and chronological (actual) age [17,18]. This is especially relevant for young people, where the impact of modifiable risk factors (e.g., smoking) on long-term CVD risk could be masked by their chronological age [17]. The Canadian Cardiovascular Society recommends the use of the FRS for the management of dyslipidemia and prevention of CVD in adults aged 30 years old and above [19]. It provides an updated score sheet with pre-defined cut-off levels for FRS risk factors to estimate the 10-year CVD risk and to quantify the CVD risk by predicting individual’s heart age [20].

Limited studies have specifically examined the relationship of diet with 10-year CVD risk and heart age using the FRS among Canadians, and none were based on the healthy eating recommendations of 2019 CFG/CDG. A Food Choices Assessment Score (FCAS) was previously developed and evaluated for alignment with the 2019 CFG/CDG recommendations, for the use in population-level dietary assessment using a food frequency questionnaires (FFQ) as the dietary assessment method [21] (S1 Table in S1 File). The FCAS showed evidence of content and construct validity by capturing the key recommendations of 2019 CFG/CDG, distinguishing between populations with known differences in their diet quality, and demonstrating a high correlation with another healthy dietary pattern score [21], also showed inverse associations with abnormal cardiometabolic risk factors [22]. However, its relationship with 10-year CVD risk and heart age among Canadians was not explored. Therefore, the aim of this study is to investigate the association between food choices aligned with 2019 CFG/CDG, measured by the FCAS, and each of the estimated 10-year CVD risk and heart age, evaluated by the Canadian Cardiovascular Society FRS, among Canadian adults.

## Methods

### Study design

The present study is a secondary analysis of the Canadian Health Measures Survey (CHMS) from 2016 to 2019 (cycles 5 & 6) data. The CHMS is a nationally representative cross-sectional survey of Canadian population aged 3–79 years old conducted by Statistics Canada in collaboration with Health Canada [23,24]. It excludes full-time members of the Canadian Forces, people living in the three territories, on reserves and settlements in the provinces, institutionalized populations, and residents of certain remote regions. Participation in the CHMS is voluntary and written consent was obtained from participants before data collection [23,24]. More details about the CHMS cycles 5 and 6 have been described elsewhere [25,26]. Briefly, the survey consists of two steps of data collection. Data on socio-demographic/economic status, chronic conditions, medication usage, smoking habits, and dietary assessment using a non-quantitative food frequency questionnaire were collected during a household interview. Data on anthropometric, physiological measures (e.g., blood pressure), and blood and urine samples were collected at the CHMS Mobile Examination Centres (MEC). Participants’ physical activities were measured using an Actical accelerometer (Philips Respironics) provided at the MEC [23,24]. For the purpose of this study, participants aged less than 30 years, participants with heart disease, pregnant women, participants with missing food frequency consumption for the FCAS food categories, participants with missing covariates (except for household income and physical activity), and participants with missing data required for calculating the FRS were excluded from the analysis (S4 Figure in S1 File). Ethics approval were obtained for all processes of the CHMS from Health Canada Research Ethics Board [23,24].

### Dietary choices assessment

Dietary intake data from the FFQ of the CHMS cycles 5 and 6 was evaluated for Canadian adults using the FCAS, which was previously developed to measure the alignment of dietary choices with 2019 CFG/CDG [21]. The FCAS consists of 9 components and sub-components, with a maximum total score of 80 points [21] (S1 Table in S1 File).

### Estimation of 10-year CVD risk and heart age

The FRS was used to estimate the 10-year CVD risk according to the Canadian Cardiovascular Society risk score criteria, which are specified for adults (≥30 years old) [20]. The FRS was calculated based on age, sex and various risk factors according to the corresponding cutoffs provided by the Canadian Cardiovascular Society score sheet. Risk factors included total cholesterol (TC) level, high density lipoprotein cholesterol (HDL-C) level, systolic blood pressure (SPB), treatment for hypertension, smoking status (daily/occasional vs. non-smoker), and the presence of diabetes (self-reported or glycated hemoglobin ≥ 6.5% [27]) [20]. Modified FRS was not estimated as the data on CVD family history was not available in the CHMS cycles 5 and 6. Ten-year CVD risk in percentage (%) was determined by the total points of the FRS and then categorized for the purpose of this study into two levels of low to intermediate risk (< 20%), and high risk (≥ 20%) of developing CVD within 10 years [28].

Heart age was estimated based on the Canadian Cardiovascular Society sex-specific FRS sheet [20]. Heart age difference was determined as heart age minus chronological (actual) age. Unhealthy heart age difference (i.e., heart age > chronological age) indicated a higher CVD risk for individuals’ chronological (actual) age [18].

### Sociodemographic and lifestyle characteristics (covariates)

Age, sex, body mass index (BMI), race/ethnicity, education level, household income, physical activity, and alcohol consumption were included in the analyses as potential covariates based on similar studies that assessed the relationship between dietary patterns or scores and 10-year CVD risk and heart age [18,28–30]. Race/ethnicity was categorized and combined, due to few numbers of participants available in various sub-groups, into 3 groups: White, East/South/Southeast Asian, and Other including (Black, West Asian, Arab, Latin American, Indigenous, multiracial, and other/unspecified groups) according to the Canadian Institutes of Health Information [31]. Participants’ education level was categorized into 3 levels (less than secondary school graduation, secondary school graduation, post-secondary graduation) based on the CHMS categorization. Household income was categorized based on quintiles, as recommended by Statistics Canada CHMS user guides [23,24], into 3 levels (high household income for the highest quintile, low for the lowest quintile, and middle for the remain quintiles). Household income was adjusted for household size according to the Statistics Canada standard [32]. Missing household income data were imputed by Statistics Canada [23,24]. Daily moderate to vigorous physical activity (MVPA) in minutes was dichotomized into sufficient (at least 150 min of MVPA/week) or not sufficient [14,33,34]. Due to important number of participants with invalid MVPA measures (25.6%), they were grouped in a third category of MVPA as invalid [35]. Lipid lowering medications were also included as potential covariates (sensitivity analysis). The CHMS medication file includes participants’ reported medications using the Anatomical Therapeutic Chemical (ATC) code. Medications with C10A and C10B codes were identified as lipid-lowering medications for sensitivity analysis [23,24,36].

### Data analysis

Descriptive statistics were conducted using chi-square tests (PROC SURVEYFREQ) to present the sociodemographic, lifestyle, 10-year CVD risk categories and heart age difference outcomes across the FCAS quintiles among Canadian adults. Mean (± SE) of the FCAS and the heart age difference across the FCAS quintiles were calculated using ANOVA test (PROC SURVEYREG).

The association between each of high risk (≥ 20%) of estimated 10-year CVD risk and unhealthy heart age difference (i.e., heart age > chronological age), as dependent variable, and FCAS quintiles were examined using multiple logistic regression (PROC SURVEYLOGISTIC). The models were adjusted for potential confounding variables, including socio-demographic/economic factors (age, sex, race/ethnicity, education level and household income), lifestyle factors (alcohol consumption and physical activity), and an anthropometric factor (BMI), as these are important factors that influence diet and CVD risk [18,28–30]. Model 1 adjusted for age, sex, education level, household income, race/ethnicity. Model 2 adjusted for variables in model 1, BMI, alcohol consumption, and physical activity. This incremental adjustment strategy helps to assess the influence of the socioeconomic and lifestyle factors on the association of the FCAS and the 10-year CVD risk and heart age, as Canadians having higher education level and/or household income were associated with higher FCAS [22]. A directed acyclic graph (S5 Figure in S1 File) illustrates our hypothesized causal structure, justifying covariate selection. Smoking, the presence of hypertension or diabetes, and the use of antihypertension or diabetes medications were part of the estimated 10-year CVD risk and heart age outcomes, so they were not included in the regression models to avoid overadjustment [18]. Model 3 adjusted for model 2, and the use of lipid lowering medications as a potential confounder (sensitivity analysis). To obtain the *P* trend, as a sequential test of the quintiles of the FCAS, the median of the FCAS in each quintile was used as a continuous variable in the logistic regression models [28,29].

To validate the dose-response association of the FCAS with the outcomes, the multiple logistic regression analyses (for models 1, 2 and 3) were also performed using the FCAS as a continuous variable [37] (sensitivity analyses).

All statistical analyses were conducted using SAS 9.4 (SAS Institute) at the Toronto Research Data Centre (RDC) in accordance with Statistics Canada confidentiality rules. Data from cycles 5 and 6 were combined, with a degree of freedom equal to 22 [38]. Sample weights provided by Statistics Canada were applied to produce population-level estimates that account for the complex sampling design of the CHMS. Variance estimation was performed using the bootstrap method with 500 replicate weights. All confidence intervals, coefficients of variation, and standard errors were calculated using these bootstrap replicate weights, as recommended by Statistics Canada [23,24].

## Results

The analysis included 5,111 adult participants, aged 30 years and above without heart disease, from the CHMS (cycles 5 and 6) representing 20,267,400 Canadian adults. Table 1 presents the weighted sociodemographic/economic, lifestyle characteristics, estimated 10-year CVD risk, and heart age difference of the study participants across the FCAS quintiles. The percentage of Canadian females who had their FCAS in the highest quintile (Q5) (healthiest) (26.6%) was higher compared to males (13.1%). Around (22%) of adults (>50 years) had their FCAS in the Q5 (healthiest), compared to (18%) of younger adults (30−50 years). Higher percentage of Canadians had their FCAS in the Q5 (healthiest) with post-secondary school education (22.9%), compared to secondary (13.6%) and less than secondary school education level (8.3%). Canadians with high risk (≥ 20%) of estimated 10-year CVD risk were more prevalent in the Q1 (unhealthy) of the FCAS (25.3%) with small percentage (14.9%) had their FCAS in the Q5 (healthiest), while Canadians with lower risk of estimated 10-year CVD were equally distributed across the FCAS quintiles. Canadians with unhealthy heart age (heart age > chronological age) were more prevalent in the Q1 (unhealthy) of the FCAS (26.1%), compared to healthy heart age (16.3%). Canadians who had their FCAS in the Q1 (unhealthy) had higher heart age than their chronological age (unhealthy age difference) (2.0 ± 0.7 years) compared to those in the Q5 (healthiest) of the FCAS with lower heart age than their chronological age (healthy age difference) (−2.8 ± 0.7 years).

**Table 1:** Weighted sociodemographic/economic and lifestyle characteristics of Canadian adults (≥30 years) without heart disease from the CHMS (cycle 5 & 6; *n* = 5,111) across the FCAS quintile categories.

Characteristics	Percentage (%)/mean^1^	Q1 (unhealthy) (%)	Q2 (%)	Q3 (%)	Q4 (%)	Q5 (healthiest) (%)
**FCAS (out of 80) (mean ± SE)**	30.5 ± 0.4	14.8 ± 0.3	23.8 ± 0.1	30.2 ± 0.1	36.7 ± 0.1	47.1 ± 0.3
**Sex**						
Males	48.7	27.2	23.5	21.0	15.2	13.1
Females	51.3	13.1	16.7	19.0	24.6	26.6
**Age (years)**						
30-50	50.6	23.3	21.4	19.8	17.5	18.0
51-79	49.4	16.6	18.7	20.2	22.5	22.0
**BMI (kg/m**^2^)						
Normal (<25.0)	36.7	17.6	18.9	18.7	20.9	23.9
Overweight (25.0–29.9)	37.3	18.4	21.9	20.2	20.1	19.4
Obesity (≥30.0)	26.0	25.7	18.8	21.6	18.4	15.5
**Education level**						
Less than secondary school	8.1	40.2	20.1^E^	22.2^E^	9.2^E^	8.3^E^
Secondary school	18.1	27.1	22.2	16.5	20.6	13.6
Post-secondary school	73.8	16.0	19.5	20.6	21.0	22.9
**Household Income** ^2^						
Low (1^st^ quintile)	17.3	27.4	22.2	13.6	16.8	20.0
Medium (2^nd^,3^rd^, 4^th^ quintiles)	57.9	20.9	19.3	20.7	19.7	19.4
High (5^th^ quintile)	24.8	12.5	20.1	22.8	22.9	21.7
**Race/ethnicity**						
Other^3^	12.3	22.6	16.4^E^	20.2^E^	22.0	18.8
White	69.7	20.2	19.9	21.3	21.1	17.5
Asian^4^	18.0	16.9	23.1	15.0	14.3	30.7
**Smoking status**						
Current smoker^5^	14.9	35.7	18.8	20.1	13.0^E^	12.4^E^
Non-smoker	85.1	17.2	20.2	20.0	21.2	21.4
**Alcohol consumption**						
Irregular/never-drinker^6^	31.3	22.8	20.9	17.6	14.9	23.8
Regular drinker	68.7	19.0	19.6	21.1	22.0	18.3
**Physical activity (moderate-to-vigorous)**						
Insufficient	41.0	20.5	20.5	21.0	19.8	18.2
Sufficient^7^	33.4	16.2	17.1	21.4	22.3	23.0
Not Valid^8^	25.6	24.0	23.1	16.5	17.3	19.1
**Self-reported lipid lowering medications use**						
No	78.5	20.6	20.9	19.2	19.8	19.5
Yes	21.5	17.5	17.0	22.5	20.4	22.6
**Estimated 10-year CVD risk**						
Low to intermediate (< 20%)	89.0	19.4	19.6	20.1	20.3	20.6
High (≥ 20%)	11.0	25.3	24.0	18.7	17.1	14.9^E^
**Heart age difference**						
Healthy (heart age ≤ chronological age)	62.3	16.3	18.9	20.6	21.3	22.9
Unhealthy (heart age > chronological age)	37.7	26.1	22.0	18.9	17.7	15.3
**Heart age difference (heart age – chronological age) (mean ± SE)**	−0.6 ± 0.7	2.0 ± 0.7	0.2 ± 0.9	−0.8 ± 0.7	−1.8 ± 0.7	−2.8 ± 0.7

Data Source = Statistics Canada, Canadian Health Measures Survey, cycles (5 and 6, 2016–2019); BMI = body mass index; CHMS = Canadian Health Measures Survey; FCAS = Food Choices Assessment Score; FFQ = Food frequency questionnaire; *n* = sample size; Q = quintile; SE = standard error.

^E^ High sampling variability (16.6 ≥ coefficient of variation ≤ 33.3); estimates should be interpreted with caution according to Statistics Canada.

^1^ Weighted percentage or mean (estimated using bootstrap 500 replicate weights provided by Statistics Canada) of Canadian adults (≥30 years old) without heart disease in each FCAS quintile category.

^2^ Adjusted according to Statistics Canada by dividing the total household income by the square root of the number of household size, and categorized into quintiles (1^st^ quintile as low income, 2^nd^, 3^rd^, 4^th^ as medium income, and 5^th^ as high income).

^3^ Including Black, West Asian, Arab, Latin American, Indigenous, multiracial, and other/unspecified groups.

^4^ Including East/South/Southeast Asian (Korean, Filipino, Japanese, Chinese, South Asian, Southeast Asian).

^5^ Occasionally or daily smoker.

^6^ Did not drink at all or drank less than once per month.

^7^ Accumulation of at least 150 minutes/week of moderate-to-vigorous physical activity.

^8^ Less than 4 valid days (at least 10 hours/day) of accelerometer wear time.

Canadian adults who had their FCAS in the Q5 (healthiest) were associated with 62% lower odds of high risk (≥ 20%) of estimated 10-year CVD risk (OR: 0.38; 95%CI: 0.19, 0.76) (*P*_trend_ = 0.009) relative to those in the Q1 (unhealthy) of the FCAS (model 1) (Table 2). This association remained with further adjustment for BMI, physical activity, and alcohol consumption (OR: 0.45; 95%CI: 0.23, 0.90) (*P*_trend_ = 0.011) (model 2).

**Table 2:** Weighted multivariable-adjusted odds ratio (OR) and 95% confidence intervals (CI) of high risk (≥ 20%) of estimated 10-year cardiovascular disease (CVD) risk according to FCAS quintiles among Canadian adults (≥30 years without heart disease; unweighted *n* = 5,111) from the CHMS (cycle 5 & 6).

	FCAS Quintiles (Q)^1^	Model 1^3^			Model 2^4^		
		**OR**^2^ **(95%CI)**	***P*-value**	***P***_**trend**_^5^	**OR (95%CI)**	***P*-value**	***P***_**trend**_^5^
High risk (≥ 20%) of estimated 10-year CVD risk	Q1 (unhealthy)	Ref.		**0.005**	Ref.		**0.011**
	Q2	0.81 (0.44, 1.50)	0.49		0.87 (0.44, 1.71)	0.67	
	Q3	0.65 (0.34, 1.23)	0.17		0.75 (0.37, 1.49)	0.39	
	Q4	0.56 (0.28, 1.11)	0.09		0.63 (0.31, 1.29)	0.20	
	Q5 (Healthiest)	0.38 (0.19, 0.76)	**0.009**		0.45 (0.23, 0.90)	**0.03**	

Data Source = Statistics Canada, Canadian Health Measures Survey, cycles (5 and 6, 2016–2019); CHMS = Canadian Health Measures Survey; CI = confidence interval; CVD = cardiovascular disease; FCAS = Food Choices Assessment Score; FFQ = Food frequency questionnaire; *n* = sample size; OR= odds ratio; Q = quintile; Ref. = reference.

^1^ Mean FCAS (out of 80) in Q1: 14.8; Q2: 23.8; Q3: 30.2; Q4: 36.7; Q5: 47.1

^2^ Odds ratios from models with different covariate sets may not be directly comparable due to scale changes inherent to logistic regression

^3^ Adjusted for age, sex, race/ethnicity, education, household income.

^4^ Adjusted for age, sex, race/ethnicity, education, household income, BMI, alcohol consumption, and physical activity [including not valid physical activity (less than 4 valid days of at least 10 hours/day of accelerometer wear time) as a separate variable].

^5^ Test for overall trend was a sequential test of the quintiles of the FCAS, using the median of FCAS in each quantile as a continuous variable in the logistic regression model.

Canadians with higher quintile of the FCAS were associated with 43–66% lower odds of having unhealthy heart age difference (heart age > chronological age) (*P*_trend_ = 0.002) relative to the Q1 (unhealthy) of the FCAS (model 1) (**Table 3**). This association remained with further adjustment for BMI, physical activity, and alcohol consumption with the corresponding OR for Canadians in the Q5 (0.53; 95%CI: 0.30, 0.92) and Q4 (0.64; 95%CI: 0.41, 0.99) (*P*_trend_ = 0.02) relative to the Q1 (unhealthy) of the FCAS (model 2).

**Table 3:** Weighted multivariable-adjusted odds ratio (OR) and 95% confidence intervals (CI) of unhealthy heart age difference (heart age > chronological age) according to FCAS quintiles among Canadian adults (≥30 years without heart disease; unweighted *n* = 5,111) from the CHMS (cycle 5 & 6).

	FCAS Quintiles (Q)^1^	Model 1^3^			Model 2^4^		
		OR^2^ (95%CI)	*P*-value	*P*_trend_^5^	OR (95%CI)	*P*-value	*P*_trend_^5^
Unhealthy heart age difference (heart age > chronological age)	Q1 (unhealthy)	Ref.		**0.002**	Ref.		**0.02**
	Q2	0.72 (0.46, 1.15)	0.16		0.76 (0.46, 1.23)	0.25	
	Q3	0.57 (0.40, 0.82)	**0.004**		0.62 (0.40, 0.95)	**0.03**	
	Q4	0.54 (0.36, 0.83)	**0.006**		0.64 (0.41, 0.99)	**0.048**	
	Q5 (Healthiest)	0.44 (0.27, 0.72)	**0.002**		0.53 (0.30, 0.92)	**0.03**	

Data Source = Statistics Canada, Canadian Health Measures Survey, cycles (5 and 6, 2016–2019); CHMS = Canadian Health Measures Survey; CI = confidence interval; CVD = cardiovascular disease; FCAS = Food Choices Assessment Score; FFQ = Food frequency questionnaire; *n* = sample size; OR= odds ratio; Q = quintile; Ref. = reference.

^1^ Mean FCAS (out of 80) in Q1: 14.8; Q2: 23.8; Q3: 30.2; Q4: 36.7; Q5: 47.1

^2^ Odds ratios from models with different covariate sets may not be directly comparable due to scale changes inherent to logistic regression

^3^ Adjusted for age, sex, race/ethnicity, education, household income.

^4^ Adjusted for age, sex, race/ethnicity, education, household income, BMI, alcohol consumption, and physical activity [including not valid physical activity (less than 4 valid days of at least 10 hours/day of accelerometer wear time) as a separate variable].

^5^ Test for overall trend was a sequential test of the quintiles of the FCAS, using the median of FCAS in each quantile as a continuous variable in the logistic regression model.

Further adjusting for the use of lipid lowering medication (model 3) showed similar results as (model 2) for the outcomes of 10-year CVD risk and heart age difference (S2 Table in S1 File).

Finding of sensitivity analyses of using the FCAS as a continuous score showed lower odds of high risk (≥ 20%) of estimated high10-year CVD (OR: 0.97; 95%CI: 0.95, 0.99) and unhealthy heart age difference (OR: 0.98; 95%CI: 0.96, 0.99) with one point increase in the FCAS (out of 80 points) (model 2) (S3 Table in S1 File).

## Discussion

The findings of this study showed that Canadian adults in the highest quintile (healthiest) of the FCAS had lower odds of having a high risk (≥ 20%) of estimated 10-year CVD risk using the Canadian Cardiovascular Society FRS. Adults in the highest quintile of the FCAS also had lower odds of having unhealthy heart age difference, compared to those in the lowest quintile (unhealthy) of the FCAS, which indicates an older heart age than their chronological (actual) age and high CVD risk according to FRS. There are limited studies that examined the relationship between diet and 10-year CVD risk among Canadians. A previous study revealed that Canadian adults consuming a dietary pattern high in carbohydrates and protein, including red and processed meat, eggs, potatoes and ice cream/frozen yogurt, associated with a high 10-year CVD risk assessed based on the 2013 American College of Cardiology/American Heart Association Guidelines [29]. This study also showed that healthy heart age difference was positively associated a “Healthy” dietary pattern and inversely associated with “Fast food” dietary pattern among Canadian adults. These dietary patterns were derived from the FFQ data of the CHMS cycles 1 and 2 (2007–2011) [29].

On the other hand, the relationship of diet with estimated CVD risk and/or heart age was assessed using other dietary scores across various populations. In agreement with the results of the present study, the Healthy Eating Index (HEI)-2015, which is an indicator of overall diet quality according to the 2015–2020 Dietary Guidelines for Americans, was inversely associated with higher estimated 10-year CVD risk, assessed by the FRS among an adult population in Iran [39]. Findings from the National Health and Nutrition Examination Surveys (NHANES) showed that adults in the highest quartile of the HEI-2015 (healthiest) had 38% lower odds of high risk (≥ 20%) of estimated 10-year CVD risk and lower heart age (−2.19 years), compared to those in the lowest quartile of the HEI-2015 (unhealthy) [28]. Among the Korean adult population, higher diet quality measured by the Korean Healthy Eating Index (KHEI) was associated with 22–27% higher odds of having healthy heart age difference (heart age < chronological age) [30]. A randomized control feeding trial of healthy adults showed that healthy dietary patterns aligned with the Dietary Approaches to Stop Hypertension (DASH) diet and a diet high in fruits and vegetables reduced the 10-year CVD risk by around 10% [7]. These diets were found to reduce the CVD risk factors, such as blood pressure, that increase the estimated 10-year CVD risk [7]. These findings reflect the outcomes of the FCAS, because the FCAS was validated against the DASH diet and was highly correlated with DASH diet score [21]. The relationship between healthy dietary patterns and the reduction of CVD incidence and mortality risk has been well documented [40,41]. An umbrella review of evidence suggested that diet quality assessed by different indices of HEI, Alternative HEI, dietary inflammatory index, Mediterranean diet, and DASH diet were associated with reduced CVD risk and mortality [40]. The Portfolio Diet Score, which is based on the plant-based portfolio dietary pattern that was designed to lower cholesterol levels, was associated with a 14% lower risk of CVD, coronary heart disease, and stroke in 3 prospective cohort studies [41]. These healthy dietary patterns share with the 2019 CFG/CDG FCAS many nutritional characteristics including high consumption of fruits, vegetables, nuts, legumes and minimal consumption of highly processed foods. The relationship between dietary choices aligned with 2019 CFG and CVD incident risk was assessed in a prospective cohort study of adult population from the UK Biobank cohort [42]. During 11-year follow up, individuals who achieved hypothetically the 90^th^ percentile (healthiest score) of the Healthy Eating Food Index (HEFI)-2019, which measures the adherence to the 2019 CFG’s recommendations based on the 24-hour dietary recall assessment method, would have a 24% lower CVD incident risk relative to those with no change in their HEFI-2019 [42]. These results support the findings of the current study about the inverse relationship between dietary choices aligned with 2019 CFG and CVD risk among Canadian adults, despite differences in the methodology of estimating CVD risk and the populations studied.

The findings should be considered in light of some limitations. The results can not be interpreted causally, due to the cross-sectional design of the study. Application of the FCAS using large-scale, long term cohort prospective data is needed to establish temporal relationships and to confirm the observed relationship with robust evidence over extended periods, including the potential relationship of the FCAS with CVD event risk and mortality. Adjusting for caloric intake was not possible due to the non-quantitative nature of the CHMS FFQ, in which serving sizes were not provided. Using an “invalid” physical activity category to account for around 25% missing data may not fully capture the effect of physical activity. Despite the importance of controlling for energy intake in epidemiological studies, it has been suggested that controlling for body weight, and physical activity, as was done here, was considerably better than poor estimation of energy intake provided by FFQ [43]. This study was also conducted using a nationally representative data of the CHMS (2016–2019), using a specific sample weight and bootstrapping method to generalize data to the Canadian population. A notable strength in this research that it is the first study to investigate the relationship between the estimated 10-year CVD risk and heart age, according to the latest FRS of the Canadian Cardiovascular Society score sheet, and the 2019 CFG/CDG FCAS among Canadians. The validity of the FCAS in measuring dietary choices quality according to the 2019 CFG/CDG was evaluated [21]. Using measured anthropometrics and physical activity for individuals as confounding variables in the regression analyses and using sensitivity analyses, to test the effects of lipid lowering medications and to verify the association outcomes using continuous FCAS, were other strengths of this study.

## Conclusions

The results of the current study indicate a strong inverse association of dietary choices aligned with 2019 CFG/CDG, as measured by the FCAS with the estimated 10-year CVD risk and unhealthy heart age (older than chronological age), according to the Canadian Cardiovascular Society FRS. These results will further support the validity of the FCAS in reflecting the recommendations of 2019 CFG/CDG to improve population health and reduce diet-related chronic diseases. These findings could have significant implications for public health. They might help in developing CVD prevention nutrition interventions aimed at reducing the CVD trend and mortality in the Canadian population; however, due to the limitations of the cross-sectional design in establishing temporal and causal relationships, larger studies are needed to evaluate prospectively the long-term relationship of the 2019 CFG/CDG FCAS and CVD at the population level. The estimated CVD risk based on the FRS helps to identify individuals at risk for primary or secondary prevention intervention. The heart age difference simplifies the absolute CVD risk concept for individuals [17]. Therefore, clinical trials may be warranted to investigate the potential benefits of these findings in clinical practice. These benefits include a possible relationship between high dietary choices quality measured by the 2019 CFG/CDG FCAS and CVD risk reduction as an intervention strategy to avoid unnecessary treatment and promote positive dietary changes.

## Supporting information

S1 File FCAS details, sensitivity analysis, and figures. This file presents FCAS information, sensitivity analysis results, and figures of the analytical sample and directed acyclic graph.
(DOCX)
