# Supplementary material for: Association of the 2019 Canada’s food guide food choices assessment score with 10-year cardiovascular disease risk and heart age in Canadian adults
Source: PLoS One. 2025 Oct 22;20(10):e0331973. doi: 10.1371/journal.pone.0331973 (PMC12543163; doi:10.1371/journal.pone.0331973)
Supplement: S1 File — This file presents FCAS information, sensitivity analysis results, and figures of the analytical sample and directed acyclic graph. (DOCX) [file pone.0331973.s001.docx]

**Supporting information**

**Association of the 2019 Canada’s Food Guide Food Choices Assessment Score with 10-year cardiovascular disease risk and heart age in Canadian adults**

Samer Hamamji^1^, Daniel Zaltz^1^, Mary R. L’Abbé^1*^

^1^Department of Nutritional Sciences, Temerty Faculty of Medicine, University of Toronto, Toronto, Ontario, Canada.

*****Corresponding author

Email: [mary.labbe@utoronto.ca](mailto:mary.labbe@utoronto.ca)

**S1 Table.** FCAS components, points allocated and scoring standards for Canadian adults based on the CHMS (cycle 5 & 6) FFQ data**^1^**.

| **FCAS components** | **Score (minimum to maximum) ^2^** | **Standard for maximum score ^3^** | **Standard for minimum score ^4^** |
| --- | --- | --- | --- |
| **FCAS components consistent with CDG - Guideline 1 and CFG snapshot with total of 40 out of 80 points ^5^** | | | |
| 1) Vegetables | 0 to 10 points | Q4 (> 20.25 times/week) | No vegetables |
| 2) Fruits | 0 to 10 points | Q4 (> 17 times/week) | No fruits |
| 3) Whole grain bread | 0 to 10 points | Q4 (> 7 times/week) | No whole grain breads |
| **Protein foods component with total 10 points and consisted of ^6^:** | | | |
| 4) Plant-based protein | 0 to 6 points | Q4 (> 7.25 times/week) | No plant-based protein |
| 5) Lean protein | 0 to 4 points | Q4 (> 16 times/week) | No lean protein |
| **FCAS components (highly processed foods) consistent with CDG - Guideline 2 with total of 40 out of 80 points ^5^** | | | |
| 6) Red and processed meat | 0 to 10 points | No red and processed meat | > Q1 (1.75 times/week) |
| 7) Processed dairy | 0 to 10 points | No processed dairy | > Q1 (2.15 times/week) |
| 8) Fries and chips | 0 to 10 points | No fries and chips | > Q1 (0.75 times/week) |
| 9) Sugary drinks | 0 to 10 points | No sugary drinks | > Q1 (0.75 times/week) |
| **Total FCAS** | **0 to 80 points** |  |  |
| Data Source= Statistics Canada, Canadian Health Measures Survey, cycles (5 and 6, 2016-2019); CDG = Canada’s Dietary Guidelines; CFG = Canada’s Food Guide; CHMS = Canadian Health Measures Survey; FCAS = Food Choices Assessment Score; FFQ = Food frequency questionnaire; *n* = sample size; Q1 = First quartile of frequency consumption; Q4 = Fourth quartile of frequency consumption.  ^1^ Hamamji. S AM, Zaltz. D, L'Abbe. M., Development and Evaluation of a Food Choices Assessment Score (FCAS) Measuring the Healthfulness of Dietary Choices According to 2019 Canada’s Food Guide/Canada’s Dietary Guidelines, using the Canadian Health Measures Survey Food Frequency Questionnaire. *Applied Physiology, Nutrition, and Metabolism*. in press.  ^2^ Scores between minimum and maximum standards were assigned proportionately.  ^3^ The fourth quartile of frequency consumption per week (above the third quartile threshold) was set as the reference standard for maximum score for FCAS components consistent with CDG - Guideline 1 and CFG snapshot recommendations of higher regular consumption of healthy food choices. No consumption was set as the reference standard for maximum score for FCAS components consistent with CDG – Guideline 2 recommendations of lower regular consumption of highly processed foods.  ^4^ No consumption was set the reference standard for minimum score for FCAS components consistent with CDG – Guideline 1 and CFG snapshot recommendations of higher regular consumption of healthy food choices. Frequency consumption per week above the first quartile threshold was set as the reference standard for minimum score for FCAS components consistent with CDG – Guideline 2 recommendations of lower regular consumption of highly processed foods.  ^5^ Total FCAS points were weighted equally (40 out of 80 points) to FCAS components consistent with CDG – Guideline 1 and CFG snapshot, and to FCAS components consistent with CDG – Guideline 2.  ^6^ Total protein food component points were weighted as 60% for plant-based sources and 40% for lean protein sources, consistent with the CFG/CDG 2019 recommendation of consuming plant-based protein source more often. | | | |

**S2 Table.** A third model of weighted multivariable-adjusted odds ratio (OR) and 95% confidence intervals (CI) of high risk (≥ 20%) of estimated 10-year cardiovascular disease (CVD) risk and unhealthy heart age difference (heart age > chronological age), according to FCAS quintiles among Canadian adults (≥30 years without heart disease; unweighted *n*= 5,111) from the CHMS (cycle 5 & 6).

|  | **FCAS Quintiles (Q)** | **Model 3**^1^ | | |
| --- | --- | --- | --- | --- |
|  |  | **OR (95%CI)** | ***P*-value** | ***P* _trend_**^2^ |
| High risk (≥ 20%) of estimated 10-year CVD risk | Q1 (unhealthy) | Ref. |  | **0.01** |
|  | Q2 | 0.75 (0.38, 1.49) | 0.68 |  |
|  | Q3 | 0.75 (0.37, 1.49) | 0.39 |  |
|  | Q4 | 0.63 (0.30, 1.30) | 0.21 |  |
|  | Q5 (Healthiest) | 0.46 (0.23, 0.90) | **0.03** |  |
| Unhealthy heart age difference (heart age > chronological age) | Q1 (unhealthy) | Ref. |  | **0.03** |
|  | Q2 | 0.76 (0.46, 1.23) | 0.252 |  |
|  | Q3 | 0.62 (0.41, 0.96) | **0.03** |  |
|  | Q4 | 0.64 (0.41, 0.99) | **0.048** |  |
|  | Q5 (Healthiest) | 0.53 (0.30, 0.92) | **0.03** |  |
| Data Source= Statistics Canada, Canadian Health Measures Survey, cycles (5 and 6, 2016-2019); CHMS = Canadian Health Measures Survey; CI = confidence interval; CVD = cardiovascular disease; FCAS = Food Choices Assessment Score; FFQ = Food frequency questionnaire; *n* = sample size; OR= odds ratio; Q= quintile; Ref.= reference.  ^1^ Adjusted for age, sex, race/ethnicity, education, household income, BMI, alcohol consumption, physical activity [including not valid physical activity (less than 4 valid days of at least 10 hours/day of accelerometer wear time) as a separate variable], and lipid lowering medication (statin).  ^2^ Test for overall trend was a sequential test of the quintiles of the FCAS, using the median of FCAS in each quantile as a continuous variable in the logistic regression model. | | | | |

**S3 Table.** Weighted multivariable-adjusted odds ratio (OR) and 95% confidence intervals (CI) of high risk (≥ 20%) of estimated 10-year cardiovascular disease (CVD) risk and unhealthy heart age difference (heart age > chronological age), based on one point increment of the FCAS (out of 80 points) among Canadian adults (≥30 years without heart disease; unweighted *n*= 5,111) from the CHMS (cycle 5 & 6).

|  | **FCAS (out of 80 points)** ^1^ | | | | | |
| --- | --- | --- | --- | --- | --- | --- |
|  | **Model 1**^2^ | | **Model 2**^3^ | | **Model 3**^4^ | |
|  | **OR (95%CI)** | ***P*-value** | **OR (95%CI)** | ***P*-value** | **OR (95%CI)** | ***P*-value** |
| High risk (≥ 20%) of estimated 10-year CVD risk | 0.97 (0.95, 0.98) | **0.001** | 0.97 (0.95, 0.99) | **0.004** | 0.97 (0.95, 0.99) | **0.005** |
| Unhealthy heart age difference (heart age > chronological age) | 0.97 (0.96, 0.99) | **0.002** | 0.98 (0.96, 0.99) | **0.02** | 0.98 (0.97, 0.99) | **0.02** |
| Data Source= Statistics Canada, Canadian Health Measures Survey, cycles (5 and 6, 2016-2019); CHMS = Canadian Health Measures Survey; CI = confidence interval; CVD = cardiovascular disease; FCAS = Food Choices Assessment Score; FFQ = Food frequency questionnaire; *n* = sample size; OR= odds ratio; Q= quintile; Ref.= reference.  ^1^ One-point increment of the FCAS as a continuous variable (out of 80 points).  ^2^ Adjusted for age, sex, race/ethnicity, education, and household income.  ^3^ Adjusted for age, sex, race/ethnicity, education, household income, BMI, alcohol consumption, and physical activity [including not valid physical activity (less than 4 valid days of at least 10 hours/day of accelerometer wear time) as a separate variable].  ^4^ Adjusted for age, sex, race/ethnicity, education, household income, BMI, alcohol consumption, physical activity [including not valid physical activity (less than 4 valid days of at least 10 hours/day of accelerometer wear time) as a separate variable], and lipid lowering medication (statin). | | | | | | |

**S4 Figure.** Analytical Sample

Excluded

- Participants under the age of 30
- Participants with heart disease
- Pregnant women
- Participants with missing data about food frequency consumption for the FCAS food categories
- Participants with missing data required for calculating the Framingham Risk Score

Population aged 3 to 79 years old in the CHMS cycles 5 and 6.

(*n* ~ 11,600)^1^

Population sample

(*n* = 5,111)^1^

^1^Sample size data are according to Statistics Canada’s data release requirements.

CHMS = Canadian Health Measures Survey; FCAS = Food Choices Assessment Score; *n* = sample size

**S5 Figure.** Directed Acyclic Graph for the Relationship of the Food Choices Assessment Score and 10-Year Cardiovascular Disease Risk and Heart Age
